# Transcriptomics differentiate two novel bioactive strains of *Paenibacillus* sp. isolated from the perennial ryegrass seed microbiome

**DOI:** 10.1038/s41598-021-94820-2

**Published:** 2021-07-30

**Authors:** Tongda Li, Ross Mann, Jatinder Kaur, German Spangenberg, Timothy Sawbridge

**Affiliations:** 1 Agriculture Victoria, AgriBio, Centre for AgriBioscience, Bundoora, VIC Australia; 2 DairyBio, Bundoora, VIC Australia; 3 School of Applied Systems Biology, La Trobe University, Bundoora, VIC Australia

**Keywords:** Bacterial genomics, Bacterial transcription

## Abstract

*Paenibacillus* species are Gram-positive bacteria that have been isolated from a diverse array of plant species and soils, with some species exhibiting plant growth-promoting (PGP) activities. Here we report two strains (S02 and S25) of a novel *Paenibacillus* sp. that were isolated from perennial ryegrass (*Lolium perenne*) seeds. Comparative genomics analyses showed this novel species was closely related to *P. polymyxa*. Genomic analyses revealed that strains S02 and S25 possess PGP genes associated with biological nitrogen fixation, phosphate solubilisation and assimilation, as well as auxin production and transportation. Moreover, secondary metabolite gene cluster analyses identified 13 clusters that are shared by both strains and three clusters unique to S25. In vitro assays demonstrated strong bioprotection activity against phytopathogens (*Colletotrichum graminicola* and *Fusarium verticillioides*), particularly for strain S02. A transcriptomics analysis evaluating nitrogen fixation activity showed both strains carry an expressed *nif* operon, but strain S02 was more active than strain S25 in nitrogen-free media. Another transcriptomics analysis evaluating the interaction of strains with *F. verticillioides* showed strain S02 had increased expression of core genes of secondary metabolite clusters (fusaricidin, paenilan, tridecaptin and polymyxin) when *F. verticillioides* was present and absent, compared to S25. Such bioactivities make strain S02 a promising candidate to be developed as a combined biofertiliser/bioprotectant.

## Introduction

Microorganisms associated with plants (collectively the plant microbiome) are one of the key factors that determine plant health and productivity^1^. Plant growth-promoting (PGP) bacteria competitively colonise plant tissues and build beneficial interactions with plant hosts by acting as biofertilisers or bioprotectants^2^, such as commercial products based on *Pseudomonas* spp. and *Rhizobium* spp. that have been utilised globally of the past 30–40 years^3^. Compared with other bacteria, the endospore-forming *Bacillus* spp. and *Paenibacillus* spp. could survive longer in soils with varying conditions such as pH, temperature and salinity, representing promising candidates for agricultural usage as biologicals^4^.

*Paenibacillus* spp. are Gram-positive, facultative anaerobic bacteria that are commonly found in soil from diverse geographic environments^4,5^. As the type species of the genus *Paenibacillus*^6^, *Paenibacillus polymyxa* inhabits the rhizosphere or root tissues of a wide range of agricultural crops like wheat and barley^7,8^, agricultural pastures like perennial ryegrass^9^ and forest trees like pine and cedar^10^. *P*. *polymyxa* has been reported to promote the growth of many plants, with significant improvements in nutrient uptake and increases in total biomass and/or seeding height^11–15^. Genomic analyses of *P. polymyxa* strains have revealed that they are capable of acting as both biofertilisers and bioprotectants^5,16–19^. As biofertilisers, *P. polymyxa* possesses genes involved in biological nitrogen (N) fixation, phosphate solubilisation and assimilation, iron assimilation and auxin production. As bioprotectants, *P. polymyxa* carries gene clusters that synthesise bioactive compounds including polymyxin and fusaricidin, as well as novel clusters of unknown functions. Furthermore, *P. polymyxa* also plays an active role in the biotechnology sector due to its ability to produce cell wall degrading enzymes and exopolysaccharides^20^. Such diverse properties have made *P. polymyxa* a prominent commercially useful PGP bacterium for sustainable agriculture^21,22^.

The microbiome of perennial ryegrass (*Lolium perenne* L. cv. Alto) has been profiled recently by our laboratory, which suggested the presence of *Paenibacillus* spp. within the community^23^. This study aimed to confirm the presence of *Paenibacillus* spp. through genomic sequencing of the seed-associated bacterial community, and to isolate strains using selective media (antibiotics and nitrogen-free media). The biological nitrogen fixation ability of the community was assessed with *nifH* PCR. The genome of the isolated strains was assembled, which was in turn used to confirm the taxonomy and confirm the presence of PGP genes and secondary metabolite gene clusters. Further assays were conducted to determine the in vitro bioprotection activities against phytopathogens and to analyse the changes in transcriptome profiles associated with biological nitrogen fixation and early stage bacteria-pathogen interactions of the isolated strains.

## Results

### Seed-associated N-fixing bacterial strain detection and isolation

The presence of bacteria containing the *nifH* gene was confirmed from the seed of perennial ryegrass, as amplicons of the expected size (~ 400 bp) were produced by the *nifH* gene PCR. Amplicons were generated using DNA extracted from a suspension of ground perennial ryegrass seeds that was serially diluted (10^0^–10^–3^), including from two of eight replicates of the 10^–2^ dilution (Supplementary Figure S1), while no amplicon was produced from all eight replicates of the 10^–3^ dilution. Amplicons were sequenced and identified as partial sequences of the *nifH* gene of *P. polymyxa* CR1 (Accession ID: CP006941.2, 1,087,670–1,088,026 bp; coverage = 97%, identity = 99%) using BLASTn search against the nt database.

Long read sequence data was also generated from the DNA of the seed suspension, and then classified by Kraken2^24^. The reads had sequence homology to multiple bacterial species including *Bacillus* spp. (high read abundance), *Pseudomonas* spp., *Massilia* spp. and *Paenibacillus* spp. (low read abundance). Despite the low *Paenibacillus* spp. read abundance, a single 110 Kb read containing the entire *P. polymyxa nif* operon (nine genes) was identified (Supplementary Figure S2), which confirmed the presence of *P. polymyxa* in the two dilutions.

The confirmation of a *P. polymyxa*-like bacterial strain in the seed suspension provided guidance regarding its isolation and purification. *P. polymyxa* produces the antibiotic polymyxin, which is biocidal against Gram-negative bacteria^25^. Supplementing media with polymyxin B aided in the isolation of the low abundant *Paenibacillus* spp. strains from the dominant bacteria. Two bacterial strains (S02 and S25) were isolated using Burk’s N-free medium supplemented with polymyxin B. Both strains are rod-shaped and Gram-positive under microscopic examination and form heaped, small- to medium-sized colonies on agar plates. Strain S02 produces white and mucoid colonies, whilst strain S25 produces translucent colonies. Both strains were stored in 15% glycerol at − 80 ℃.

### Genome sequencing, assembly and annotation

A total of 2,536,823,196 bp short reads and 13,203,686,400 bp long reads were generated for *Paenibacillus* sp. strains S02 and S25 (Supplementary Table S1). Complete circular genome sequences were produced for both strains. The genome size of *Paenibacillus* sp. S02 was 6,060,529 bp (5,310 CDSs), with a G + C content of 45.60%, while the genome size of *Paenibacillus* sp. S25 was 5,958,851 bp (5,177 CDSs), with a G + C content of 45.72% (Supplementary Table S2). There were no plasmids present in either strain.

### Phylogeny and comparative genomics

The results of the *nifH* gene PCR and the mixed culture read analysis described in “Seed-associated N-fixing bacterial strain detection and isolation” section suggested that the isolated strains were closely related to the species *P. polymyxa*. The 16S ribosomal RNA genes showed both strains were phylogenetically related to *P. polymyxa* DSM36 (GenBank Accession: NR_117732.2) with a sequence homology of 99.45% and coverage of 100%. The close relationship between the two strains and *P. polymyxa* was further supported by genome-based identifications where both strains were classified by Kraken2 as *P. polymyxa* E681 (NCBI:txid 349520).

**Figure 1:**
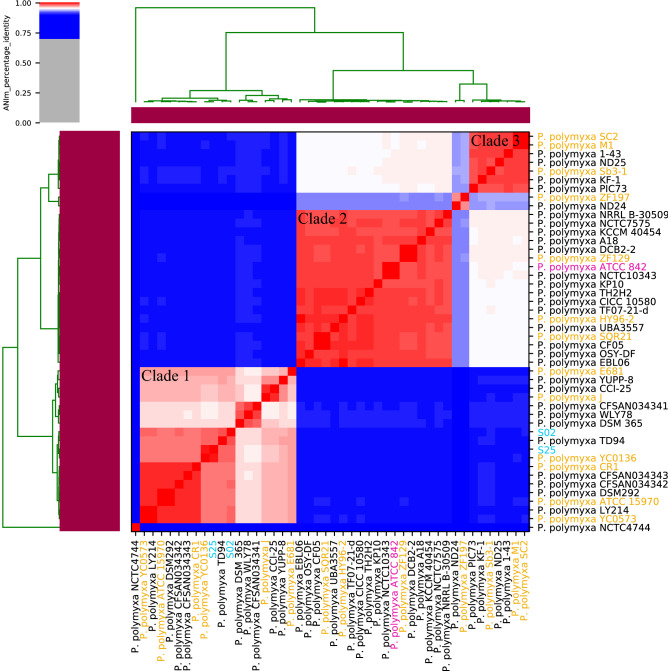
Phylogeny of *Paenibacillus* species based on a heatmap with row and column dendrograms from the ﻿average nucleotide identity (ANI) of genomes of *Paenibacillus* sp. strains S02 and S25 and 44 *P. polymyxa* strains from NCBI. Clustering across the dendrograms were based on overall genomic sequence similarity, forming three major clades and two minor clades (intra-cluster ANI > 95%). Clade 1 contained *Paenibacillus* sp. strains S02 and S25 as well as 16 known *P. polymyxa* strains. Clade 2 and 3 contained 18 and seven known *P. polymyxa* strains, respectively. *P. polymyxa* ZF197 and ND24 formed a minor clade, and *P. polymyxa* NCTC4744 formed another minor clade. Blue label: *Paenibacillus* sp. strains isolated in this study. Yellow label: *P. polymyxa* strains with complete circular genome sequences. Purple label: The type strain of *P. polymyxa.*

The average nucleotide identity (ANI) was compared between the genomes of the two isolated strains (S02 and S25) and 44 known *P. polymyxa* strains (Supplementary Table S3). The ANI dendrogram-heatmap revealed three major (Clades 1–3) and two minor clades (Fig. 1). The ANI between strains within the same clade was at least 95%. Among the three major clades, strains from Clade 2 and 3 had ANI < 95%, while Clade 1 was further separated from the other two clades (ANI < 91%). *Paenibacillus* sp. strains S02 and S25 were in Clade 1 and had an ANI of 97.78% to one another. Strain S02 was most similar to *P. polymyxa* TD94 (ANI = 98.11%) which was isolated from *Scutellaria* spp. rhizosphere^26^, while strain S25 was most similar to *P. polymyxa* YC0136 (ANI = 99.29%) which was isolated from tobacco rhizosphere^19^. Both strains were clustered with 16 known *P. polymyxa* strains isolated from various geographic regions including Asia, North America and Europe. The majority of the Clade 1 strains (14) were isolated from plant rhizosphere or soil, with the exceptions being *P. polymyxa* CCI-25 isolated from vermicompost^18^, and *P. polymyxa* J isolated from the phloem of a chilli plant. Clades 2 and 3 contained 18 and seven *P. polymyxa* strains respectively, and these strains were mainly isolated from plant rhizosphere or soil in Asia, Europe and North America. The type strain of *P. polymyxa* (ATCC 842) was placed in Clade 2. *P. polymyxa* ZF197 and ND24 formed a minor clade that had an ANI of 92.45–92.61% and 92.93–93.09% when compared to Clade 2 and 3, respectively. There was also another minor clade containing a single strain *P. polymyxa* NCTC4744 isolated from the UK, which had low ANI values (< 89%) when compared to the other strains.

**Figure 2:**
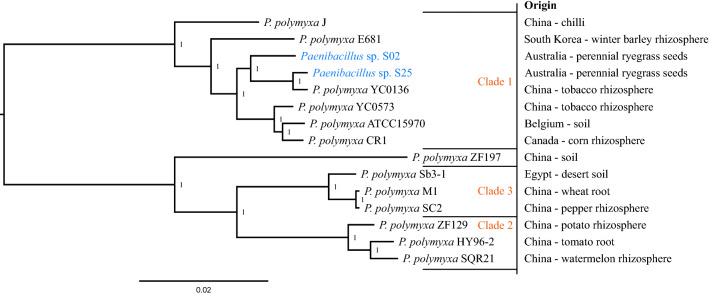
Phylogeny of *Paenibacillus* species based on a pan genome Roary analysis of strains S02 and S25 and 13 *P. polymyxa* strains with complete circular genome sequences. The maximum-likelihood tree was inferred based on 2059 genes conserved among 15 genomes. Values shown next to branches were the local support values calculated using 1,000 resamples with the Shimodaira-Hasegawa test. *Paenibacillus* sp. strains S02 and S25 clustered with other six *P. polymyxa* strains in Clade 1. Clades 2 and 3 separated from Clade 1 at the root node, and consisted of three *P. polymyxa* strains each.

A pan genome Roary^27^ analysis was conducted comparing the sequence similarity of genes shared by *Paenibacillus* sp. strains S02 and S25, along with 13 other *P. polymyxa* strains with complete genomes available on NCBI. The analysis identified 2059 shared genes by all 15 strains. A maximum-likelihood phylogenetic tree was inferred based on the sequence homology of the shared genes (Fig. 2). The topology of the tree consisted of three major clades and was consistent with the ANI analysis (Fig. 1, Clade 1–3). All clades were separated with a strong local support value (100%). Clade 1 consisted of eight strains, including the two *Paenibacillus* sp. strains (S02 and S25), and were separated from Clades 2 and 3 at the root node. Clades 2 and 3 formed adjoining clades on the same primary root node, and each had three strains. Strain ZF197 also clustered with Clade 2 and 3 but formed its own branch. Clade 1 consisted of strains from across a broad geographic range, including Asia (China, South Korea), the Pacific (Australia), North America (Canada) and Europe (Belgium), whereas Clades 2 and 3 were largely from Asia (China), except strain Sb3-1 in Clade 3 that was isolated from Egypt. All strains were either associated with plants or soil.

### Plant growth-promoting genes

The genomes of both *Paenibacillus* sp. strains (S02 and S25) were assessed for the presence of 30 plant growth-promoting (PGP) genes and were found to possess a comprehensive set of genes (Supplementary Table S4). A 10.54 Kb region containing a *nif* operon of nine genes (*nifB/H/D/K/E/N/X*, *hesA/moeB* and *nifV*) was identified, including all the genes necessary for encoding the Mo-nitrogenase (molybdenum-dependent nitrogenase) that catalyses biological nitrogen fixation^28^. In addition, 16 genes associated with phosphate solubilisation and assimilation were identified, including the glucose-1-dehydrogenase (*gcd*) gene for inorganic phosphate solubilisation^29^, the *phn* cluster of nine genes for organic phosphate (phosphonates) solubilisation^2^ and the phosphate-specific transport system of six genes for phosphate assimilation^30^. However, the gluconic acid dehydrogenase (*gad*) gene for inorganic phosphate solubilisation^29^ was not found in either of the two strains. Additionally, genes involved in indole-3-acetic acid (IAA) production and transportation were identified, including the *ipdC* gene that encodes a key enzyme in the IAA biosynthetic pathway^31^, as well as three auxin efflux carrier genes. Sequence comparison of the PGP genes between the two strains (S02 and S25) showed sequence similarity of 95.39–99.54%, while the two strains showed sequence similarity of 94.69–99.78% when compared to *P. polymyxa* strain CR1 (Supplementary Table S5).

### Secondary metabolite genes

Genes associated with secondary metabolite production were identified using antiSMASH^32^. The analyses identified 16 clusters (designated C1–C16) consisting of 13 clusters that were shared by both strains and three clusters that only strain S25 possessed (Supplementary Table S6). All clusters contained all the genes (core/additional biosynthetic genes, regulatory genes, transport-related genes and other genes) required for complete function.

Secondary metabolite gene clusters that were shared by both strains consisted of four that were identical to known clusters, including three nonribosomal peptide synthetase (Nrps) clusters (C1, fusaricidin B; C10, tridecaptin; C15, polymyxin) and one lanthipeptide cluster (C7, paenilan). The products of all four clusters have been reported to have antimicrobial bioactivities^25,33–35^. A further four clusters had partial sequence homology to known clusters included a lassopeptide cluster (C5), a Nrps cluster (C6), a Nrps/transAT-polyketide synthase (PKS) cluster (C11) and a Nrps/Type III (T3) PKS/transAT-PKS cluster (C14), which had homology to paeninodin, marthiapeptide A, paenilipoheptin and aurantinin B/C/D, respectively. Among these four clusters, cluster C11 had the highest similarity (S02, 73%; S25, 76%) to a known cluster of *P. polymyxa* E681 that produces paenilipoheptin^36^. There were also five clusters that appear novel based on sequence homology, including a siderophore cluster (C2), a bacteriocin cluster (C3), a Nrps/transAT-PKS cluster (C4), a Nrps-like cluster (C9) and a phosphonate cluster (C16).

*Paenibacillus* sp. S25 had three unique secondary metabolite gene clusters that were missing in the genome of *Paenibacillus* sp. S02, including a lanthipeptide cluster (C8) and two novel Nrps clusters (C12, C13). While the two Nrps clusters appear novel based on sequence homology, the lanthipeptide cluster had a similarity of 71% to a known paenicidin B cluster, which was a novel lantibiotic peptide active against Gram-positive bacteria produced by *Paenibacillus terrae*^35^.

Secondary metabolite gene cluster analyses were also conducted using the 13 *P. polymyxa* strains with complete circular genome sequences (Fig. 1, yellow labels), and their presence was compared to *Paenibacillus* sp. strains S02 and S25. The total number of secondary metabolite gene clusters of each strain varied between 11 to 16 (Table 1). Clade-specific clusters were identified, including one associated with a novel siderophore and another associated with a novel phosphonate being exclusive to Clade 1 strains, and a nostamide A (betalactone) cluster being exclusive to Clade 2 strains. Moreover, the paenilipoheptin cluster was only identified in some Clade 1 and 3 strains but no Clade 2 strain. More than half of the identified clusters were Nrps or PKS, including a tridecaptin cluster that was shared by all strains regardless of the phylogenetic clades. The fusaricidin B cluster was identified in all strains except *P. polymyxa* CR1, and the polymyxin cluster was identified in all strains except *P. polymyxa* strains YC0573, ATCC 15,970 and CR1. Nrps/PKS clusters producing novel products were also identified in all strains. Lanthipeptide clusters producing both novel and known products were also widely distributed in all strains. Specifically, the paenilan cluster was identified in all strains except *P. polymyxa* strains J and ATCC 15,970 (Clade 1), however the paenibacillin cluster and the paenicidin A/B cluster were less common. The paenibacillin cluster was only identified in *P. polymyxa* ZF129 (Clade 2), and the paenicidin A/B cluster was only identified in all Clade 2 strains and one Clade 1 strain (*P. polymyxa* J). Other widely distributed secondary metabolite gene clusters included one novel lassopeptide cluster and one novel bacteriocin cluster.

**Table 1:**
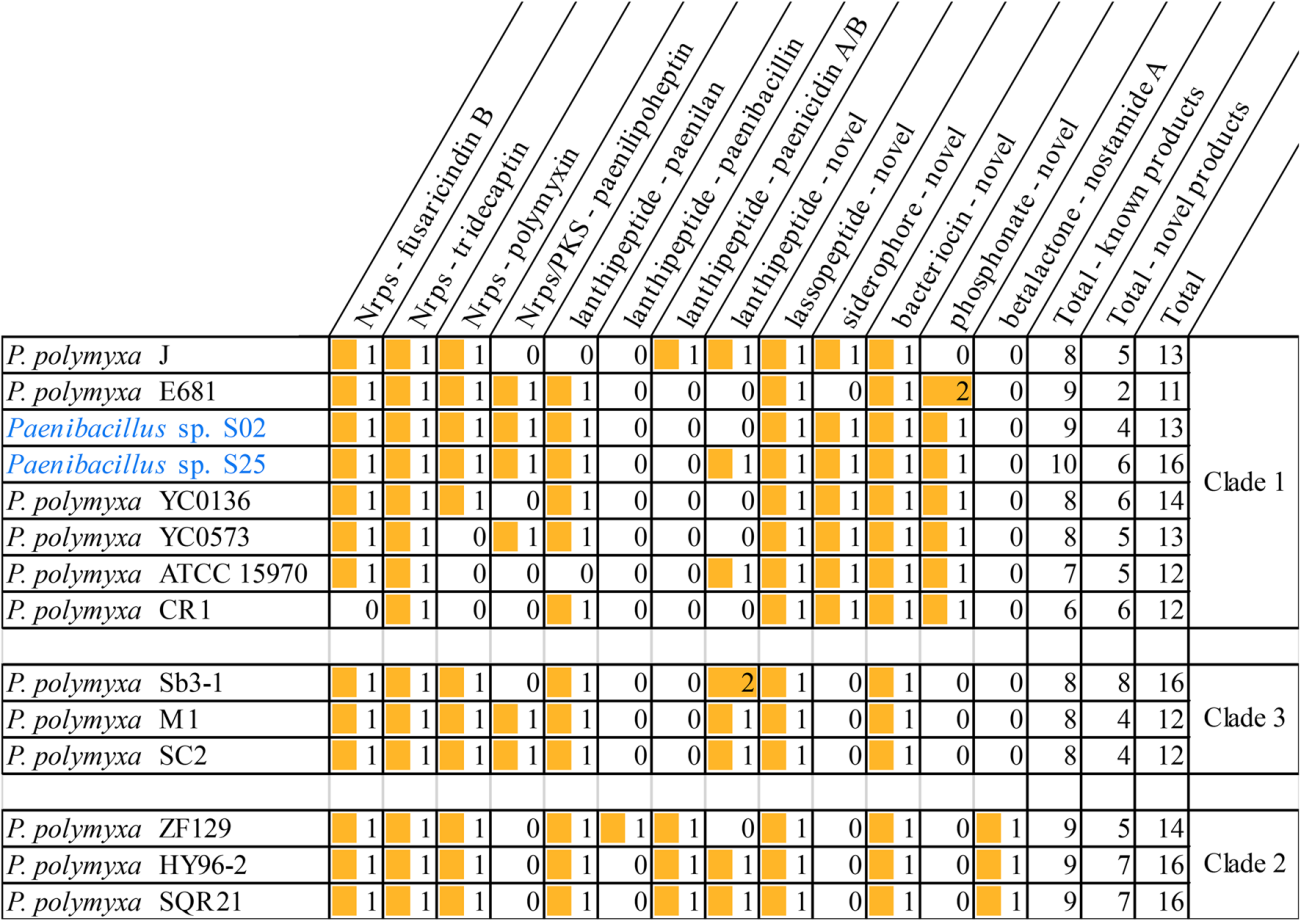
Secondary metabolite gene clusters identified in *Paenibacillus* sp. strains S02 and S25 and 13 *P. polymyxa* strains with complete circular genome sequences.

The type and product of each secondary metabolite gene cluster were shown in the first row. Numbers are the total count of each secondary metabolite gene cluster. The length of the orange bar represents the total count of each secondary metabolite gene cluster.

Novel: Similarity ≤ 70% when compared to the most similar known cluster in the antiSMASH database.

Nrps: Nonribosomal peptide synthetase.

PKS: Polyketide synthase.

Clade 1/2/3: Clades identified in phylogeny and comparative genomics study (“Phylogeny and comparative genomics” section).

### Bioprotection assay (in vitro)

A dual culture in vitro assay was established to compare the biocidal activity of *Paenibacillus* sp. strains S02 and S25 against three fungal pathogens, *Colletotrichum graminicola*, *Fusarium verticillioides* and *Microdochium nivale*. *Paenibacillus* sp. S02 significantly (*P* < 0.05) reduced the average colony diameter of the fungal pathogens *C. graminicola* and *F. verticillioides* compared to the blank control and *Paenibacillus* sp. S25 (Supplementary Table S7, Supplementary Figure S3). It reduced the growth of *C. graminicola* and *F. verticillioides* by up to 74.9% and 56.9%, respectively. *Paenibacillus* sp. S25 significantly (*P* < 0.05) reduced the growth of *F. verticillioides* by 9.6% compared to the blank control, however no similar activity was observed for *C. graminicola*. Neither of the two strains could significantly reduce the average colony diameter of *M. nivale*.

### Transcriptome sequencing and analysis—﻿N-fixation activity assay

**Figure 3:**
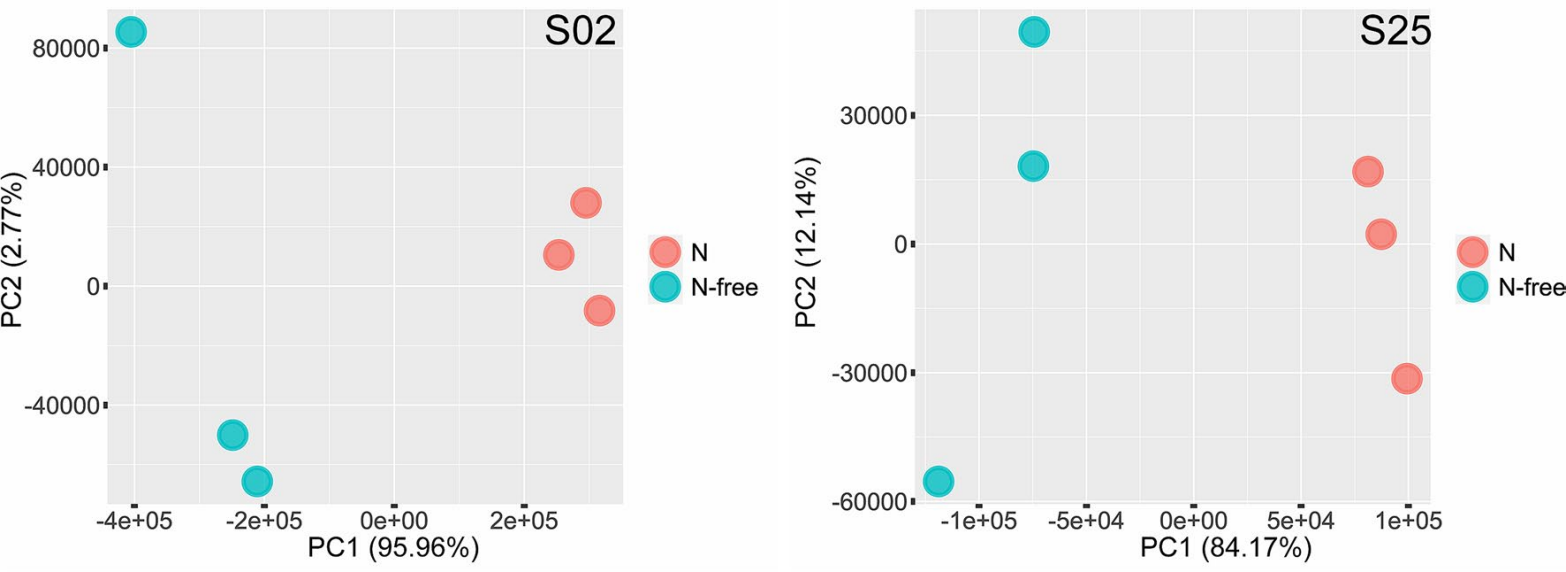
PCA plots of transcriptome profiles of *Paenibacillus* sp. strains S02 (left) and S25 (right) when grown in media with nitrogen (N) and without nitrogen (N-free) in the N-fixation activity assay. Percentage variance explained by each axis are given in brackets. Distinctive clusters indicate that the presence/absence of a nitrogen source (NH_4_Cl) in the media changed the transcriptome profiles of both strains.

A transcriptome sequencing experiment was designed to confirm the expression of the *nif* operon of *Paenibacillus* sp. strains S02 and S25 when the nitrogen source (NH_4_Cl) was removed from the media. A 150 bp PE library prepared from cDNA from strains used in the N-fixation activity assay generated an average of 13.8 million clean reads per sample. Differential gene expression (DGE) analysis successfully identified genes that were differentially expressed under different conditions in the assay. A total of 5,059 and 4,745 genes passed the abundance filter for strain S02 and S25, respectively, and were used in the subsequent DGE analysis. Among those genes, 2,467 and 2,479 genes were differentially expressed when nitrogen was removed from the media for strain S02 and S25, respectively. Biological replicates of the nitrogen treatment (+ / −) formed distinctive clusters along the PC1 axis for both strains, suggesting the presence/absence of nitrogen impacted the transcriptome profiles of both strains (Fig. 3). Specifically, when nitrogen was present in the media, the *nif* operon comprising nine genes was expressed by both strains, and there was no significant difference in expression levels of any *nif* gene between the two strains. However, the expression levels of the *nif* operon varied when nitrogen was removed from the media (Table 2). Gene expression levels when nitrogen was removed were represented as fold-changes in relation to the expression levels when nitrogen was present. The expressions of all nine genes of the *nif* operon of *Paenibacillus* sp. S02 were upregulated by 8.62–22.50 folds. For *Paenibacillus* sp. S25, the *nifB/H/D/K/E* genes were differentially expressed with 1.76- to 3.90-fold increase. The remaining four genes of the *nif* operon also had fold-changes in expression level, however they failed to be considered as differentially expressed (*q-value* < 0.05 and absolute fold-change ≥ 1.5). Such results confirmed that both strains likely carry a biologically functional *nif* operon that enables biological nitrogen fixation.

**Table 2:** Changes in expression levels of the *nif* operon of *Paenibacillus* sp. strains S02 and S25 when NH_4_Cl was removed from the media.

*nif* genes	S02	S25
	Fold-change	Fold-change
*nifB*	22.50*	2.46*
*nifH*	20.21*	3.90*
*nifD*	15.80*	2.06*
*nifK*	17.51*	2.01*
*nifE*	15.86*	1.76*
*nifN*	18.16*	1.59
*nifX*	8.62*	1.19
*hesA/moeB*	15.13*	1.56
*nifV*	11.01*	− 1.46

*Genes that were differentially expressed (*q-value* < 0.05 and absolute fold-change ≥ 1.5) when nitrogen was removed from the media.

### Transcriptome sequencing and analysis—Bacteria-pathogen interactions assay

**Figure 4:**
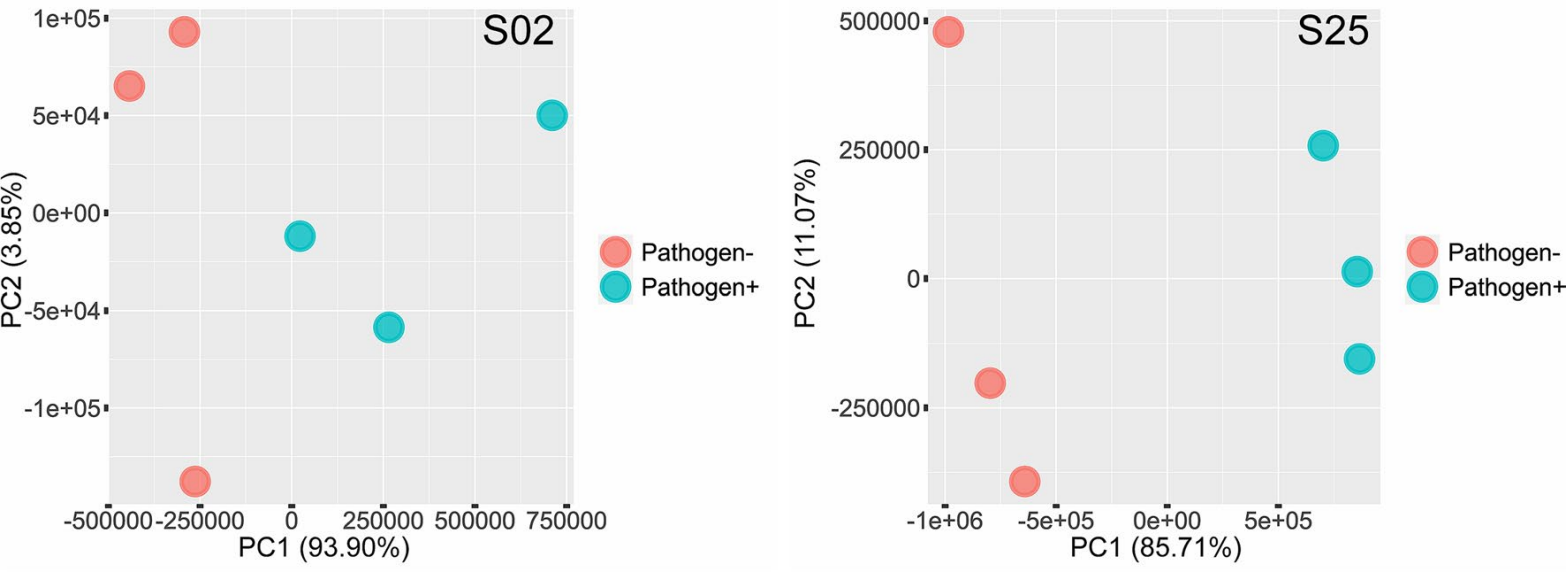
PCA plots of transcriptome profiles of *Paenibacillus* sp. strains S02 (left) and S25 (right) when grown in media with *F. verticillioides* (Pathogen+) and without *F. verticillioides* (Pathogen−) in the bacteria-pathogen interactions assay. Percentage variance explained by each axis are given in brackets. Distinctive clusters indicate that the presence/absence of *F. verticillioides* in the media changed the transcriptome profiles of strain S25 (along the PC1 axis).

A transcriptome sequencing experiment was designed to explore the early stage interactions between *Paenibacillus* sp. strains S02 and S25 and the fungal pathogen *F. verticillioides*. A 150 bp PE library prepared from cDNA from strains used in the bacteria-pathogen interactions assay generated an average of 31.1 million clean reads per sample. DGE analysis successfully identified genes that were differentially expressed under different conditions in the assay. A total of 5,201 and 4,817 genes passed the abundance filter for strain S02 and S25, respectively, and were used in the subsequent DGE analysis. Among those genes, only 61 genes were differentially expressed by strain S02 when *F. verticillioides* was present. In contrast, 2,706 genes were differentially expressed by strain S25 when *F. verticillioides* was present. Moreover, distinctive clustering of the three biological replicates based on transcriptome profiles was identified for strain S25 along the PC1 axis, but the clustering was not as evident for strain S02, particularly when the pathogen was present (Fig. 4). The Clusters of Orthologous Groups (COGs) of proteins^37^ encoded by the genes mentioned above rendered an overview of functions of those genes. For *Paenibacillus* sp. S02, 29 genes were associated with cellular processes and signalling, and 14 genes were associated with metabolism. There were also 18 genes with unknown functions. For *Paenibacillus* sp. S25, 368 genes were associated with cellular processes and signalling, and 889 genes were associated with metabolism. In addition, there were 416 genes associated with information storage and processing, 43 genes associated with multiple function groups and 990 genes with unknown functions. Given the complexity of transcriptome profiles, this study focused on the expression levels of the core biosynthetic genes of secondary metabolite gene clusters identified in “Secondary metabolite genes” section to demonstrate the early stage bacteria-pathogen interactions.

When *F. verticillioides* was absent, *Paenibacillus* sp. S02 showed increased expressions of the core biosynthetic genes of secondary metabolite gene clusters compared to *Paenibacillus* sp. S25. Gene expression levels of those genes of strain S02 were represented as fold-changes in relation to the expression levels of the corresponding homolog of each gene of strain S25 (Table 3). Among the 44 core biosynthetic genes of secondary metabolite gene clusters shared by the two strains, the majority (41) were expressed in increased levels ranging from 1.92- to 486.58-fold increase, including the four clusters that produce known antimicrobial compounds (C1, C7, C10 and C15). Only one cluster had a decreased expression, with C9 exhibiting a 7.42-fold decrease in the expression level. Two core biosynthetic genes of cluster C4 were not differentially expressed when comparing the two strains.

**Table 3:** Fold-changes in expression levels of the core biosynthetic genes of secondary metabolite gene clusters of strain S02 compared to strain S25 when *F. verticillioides* was absent.

ID	Type	Most similar known cluster (similarity)	*Paenibacillus* sp. S02	
			Gene ID	Fold-change
C1	Nrps	**Fusaricidin B (100%)**	KAI36_00078	486.58*
			KAI36_00083	20.36*
C2	Siderophore	–	KAI36_00955	11.71*
			KAI36_00956	13.93*
			KAI36_00959	4.64*
C3	Bacteriocin	–	KAI36_01103	46.95*
C4	Nrps transAT-PKS	–	KAI36_01166	18.88*
			KAI36_01170	2.43*
			KAI36_01172	1.93*
			KAI36_01173	3.31*
			KAI36_01175	2.35*
			KAI36_01176	2.30*
			KAI36_01178	2.27*
			KAI36_01179	1.92*
			KAI36_01180	– 1.25
			KAI36_01181	1.27
C5	Lassopeptide	Paeninodin (40%)	KAI36_01236	134.38*
			KAI36_01240	37.82*
C6	Nrps	Marthiapeptide A (33%)	KAI36_01339	239.45*
			KAI36_01340	159.93*
			KAI36_01341	119.29*
C7	Lanthipeptide	**Paenilan (100%)**	KAI36_01558	60.94*
			KAI36_01560	271.12*
			KAI36_01562	69.08*
C9	Nrps-like	–	KAI36_01944	– 7.42*
C10	Nrps	**Tridecaptin (100%)**	KAI36_02333	7.74*
			KAI36_02334	32.36*
C11	Nrps transAT-PKS	Paenilipoheptin (S02, 73%; S25, 76%)	KAI36_02506	17.20*
			KAI36_02507	29.10*
			KAI36_02508	23.54*
			KAI36_02509	22.65*
			KAI36_02510	10.63*
C14	Nrps T3PKS transAT-PKS	Aurantinin B/C/D (35%)	KAI36_03362	118.38*
			KAI36_03363	46.83*
			KAI36_03365	125.65*
			KAI36_03366	98.69*
			KAI36_03367	31.92*
			KAI36_03368	77.19*
			KAI36_03371	134.46*
			KAI36_03372	113.90*
C15	Nrps	**Polymyxin (100%)**	KAI36_04684	9.61*
			KAI36_04687	8.61*
			KAI36_04688	9.86*
C16	Phosphonate	–	KAI36_05277	15.10*

Clusters in bold: Known antimicrobial compounds.

*Genes that were differentially expressed (*q-value* < 0.05 and absolute fold-change ≥ 1.5) when comparing the two strains.

The presence of *F. verticillioides* had different effects in expressions of the core biosynthetic genes of secondary metabolite gene clusters on the two strains. Gene expression levels when *F. verticillioides* was present were represented as fold-changes in relation to the expression levels when *F. verticillioides* was absent (Table 4). For *Paenibacillus* sp. S02, only three of 44 core biosynthetic genes of secondary metabolites were differentially expressed, while for *Paenibacillus* sp. S25, 32 of 51 genes were differentially expressed. Despite clusters C1, C7, C10 and C15, which produce known antimicrobial compounds, being shared by both strains, the expression levels of core biosynthetic genes of secondary metabolite varied. For instance, the four genes of the polymyxin cluster (C15) were not differentially expressed by strain S02 but were differently expressed with 1.45- to 2.61-fold decrease by strain S25. Strain S25 had a gene of the fusaricidin B cluster (C1) that was differentially expressed with a 5.21-fold decrease, and two genes of the tridecaptin cluster (C10) that were differentially expressed (one with a 1.92-fold decrease and the other with a 1.79-fold increase), whereas these gene clusters were not affected in strain S02. The three core biosynthetic genes of the paenilan cluster (C7) were not differentially expressed in either strain. As for the four clusters that had the best matches in the antiSMASH gene cluster database (C5, C6, C11 and C14), although the core genes had differing levels of fold-change, the trend of changes (i.e. upregulated or downregulated) were consistent between strains. Similar consistencies were also observed from the core biosynthetic genes of three shared novel clusters (C4, C9 and C16). Furthermore, the core gene of the bacteriocin cluster (C3) was not differentially expressed in either strain. However, two genes of the siderophore cluster (C2) of strain S25 were differentially expressed with a 2.06- and 2.60-fold increase, respectively, unlike strain S02. Moreover, amongst the three novel clusters (C8, C12 and C13) that were unique to strain S25, two (C12 and C13) had differentially expressed core genes.

**Table 4:** Fold-changes in expression levels of the core biosynthetic genes of secondary metabolite gene clusters identify in *Paenibacillus* sp. strains S02 and S25 when *F. verticillioides* was present.

ID	Type	Most similar known cluster (similarity)	*Paenibacillus* sp. S02		*Paenibacillus* sp. S25	
			Gene ID	Fold-change	Gene ID	Fold-change
C1	Nrps	**Fusaricidin B (100%)**	KAI36_00078	− 1.28	KAI37_00078	− 1.50
			KAI36_00083	− 1.36	KAI37_00083	− 5.21*
C2	Siderophore	–	KAI36_00955	− 1.23	KAI37_00927	2.06*
			KAI36_00956	− 1.15	KAI37_00928	2.60*
			KAI36_00959	− 1.12	KAI37_00931	1.04
C3	Bacteriocin	–	KAI36_01103	1.10	KAI37_01049	− 1.19
C4	Nrps transAT− PKS	–	KAI36_01166	− 1.25	KAI37_01130	− 1.17
			KAI36_01170	− 1.89*	KAI37_01134	− 1.08
			KAI36_01172	− 1.54	KAI37_01136	− 1.43
			KAI36_01173	− 1.65*	KAI37_01137	− 1.49
			KAI36_01175	− 1.64	KAI37_01139	− 1.52
			KAI36_01176	− 1.48	KAI37_01140	− 1.56
			KAI36_01178	− 1.41	KAI37_01142	− 1.51*
			KAI36_01179	− 1.40	KAI37_01143	− 1.55
			KAI36_01180	− 1.43	KAI37_01144	− 2.34*
			KAI36_01181	− 1.33	KAI37_01145	− 1.90*
C5	Lassopeptide	Paeninodin (40%)	KAI36_01236	− 1.20	KAI37_01200	− 3.63*
			KAI36_01240	− 1.30	KAI37_01204	− 2.07
C6	Nrps	Marthiapeptide A (33%)	KAI36_01339	1.19	KAI37_01293	4.34*
			KAI36_01340	1.15	KAI37_01294	2.43*
			KAI36_01341	1.12	KAI37_01295	2.02*
C7	Lanthipeptide	**Paenilan (100%)**	KAI36_01558	− 1.04	KAI37_01518	1.32
			KAI36_01560	− 1.00	KAI37_01520	1.10
			KAI36_01562	1.02	KAI37_01522	1.18
C8	Lanthipeptide	**Paenicidin B (71%)**			KAI37_01661	− 1.73
					KAI37_01663	− 1.19
C9	Nrps− like	–	KAI36_01944	1.81*	KAI37_01854	2.07*
C10	Nrps	**Tridecaptin (100%)**	KAI36_02333	1.02	KAI37_02322	− 1.92*
			KAI36_02334	1.00	KAI37_02323	1.79*
C11	Nrps transAT− PKS	Paenilipoheptin (S02, 73%; S25, 76%)	KAI36_02506	1.14	KAI37_02476	15.43*
			KAI36_02507	1.16	KAI37_02477	23.42*
			KAI36_02508	1.16	KAI37_02478	11.77*
			KAI36_02509	1.21	KAI37_02479	7.92*
			KAI36_02510	1.11	KAI37_02480	3.23*
C12	Nrps	–			KAI37_02516	-1.91*
C13	Nrps betalactone	–			KAI37_02623	1.50*
					KAI37_02624	2.02*
					KAI37_02633	2.10*
C14	Nrps T3PKS transAT-PKS	Aurantinin B/C/D (35%)	KAI36_03362	− 1.29	KAI37_03372	1.71
			KAI36_03363	− 1.35	KAI37_03373	− 1.78
			KAI36_03365	− 1.32	KAI37_03375	− 1.66*
			KAI36_03366	− 1.34	KAI37_03376	− 1.82*
			KAI36_03367	− 1.26	KAI37_03377	− 2.00*
			KAI36_03368	− 1.05	KAI37_03378	− 1.78*
			KAI36_03371	− 1.05	KAI37_03381	N/A
			KAI36_03372	− 1.03	KAI37_03382	− 4.68*
C15	Nrps	**Polymyxin (100%)**	KAI36_04684	− 1.02	KAI37_04566	− 2.61*
					KAI37_04567	− 2.33*
			KAI36_04687	1.10	KAI37_04570	− 1.45*
			KAI36_04688	1.12	KAI37_04571	− 1.92*
C16	Phosphonate	–	KAI36_05277	− 1.38	KAI37_05154	− 1.77*

Clusters in bold: Known antimicrobial compounds.

N/A: Genes that didn’t pass the abundance filter described in “Transcriptome analysis” section.

*: Genes that were differentially expressed (*q-value* < 0.05 and absolute fold-change ≥ 1.5) when *F. verticillioides* was present.

Fungal transcripts of *F. verticillioides* were also assessed as a part of the bacteria-pathogen interactions assay. Transcript quantification using the transcriptome sequences of *F. verticillioides* 7600 as the reference reflected the differences in bioprotection activities between the two strains (Supplementary Table S8). The percentage of mapped reads in the treated samples for *Paenibacillus* sp. S02, which showed stronger bioprotection activities in the in vitro assay (“Bioprotection assay (in vitro)” section), was much lower than that for *Paenibacillus* sp. S25. Furthermore, amongst the treated samples for *Paenibacillus* sp. S02, the percentage of mapped reads of the replicate 1 (S02_treated_1) was even comparable to the untreated samples.

## Discussion

### Isolation and identification of novel *Paenibacillus* sp. strains

Two N-fixing bacterial strains were isolated in this study using a combined approach that took advantage of both microbiological techniques (N-free medium) and genomic resources (sequencing). Various N-free media have been widely used to isolate N-fixing microorganisms from natural environments on the basis that nitrogen is required by all organisms to survive^28,38^. However, being able to grow in N-free media does not necessarily guarantee the ability to fix atmospheric nitrogen by a microorganism. For instance, *Streptomyces thermoautotrophicus* UBT1 was initially reported as a N-fixing bacterium^39^, however further analysis revealed that the solidifying agent used to prepare the N-free medium contained 0.095% nitrogen content^40^. Hence, instead of being a N-fixing bacterium, this strain was a N-scavenger that could utilise nitrogen present in ultra-low concentrations. Furthermore, even if the selective medium is N-free, those scavengers could still grow in combination with other N-fixing microorganisms present, hence hindering the isolation process, which was observed in this study. The N-free medium and the *nifH* gene PCR used in this study confirmed the presence of N-fixing bacteria in the culture. Moreover, the sequencing results provided possible identities of the N-fixing bacteria, which greatly assisted the isolations of the two strains (S02 and S25). The long-read sequencing data of dilutions confirmed the presence of *Paenibacillus* spp. and also revealed the possible identity of other bacterial species in dilutions, leading to the addition of polymyxin B, which removed all N-scavengers in the culture.

Taxonomic identification of bacterial species based on the 16S ribosomal RNA and whole genome sequence homology suggested that the two strains used in this study (S02 and S25) were closely related to *P. polymyxa*. Furthermore, the ANI analysis showed that the two strains were not genetically identical to any of the known *P. polymyxa* strains, and hence represent two novel strains of *Paenibacillus* spp. There are currently (27/07/2020) genome sequences of 56 *P. polymyxa* strains publicly available on NCBI, none of which was originally isolated from Australia. Therefore, this is the first study that reported Australian strains of this species. *P. polymyxa* has been extensively studied, and some strains have been developed as commercially available biopesticides or biofertilisers^5^. The two strains (S20 and S05) reported by this study will be of great benefit to the local application of this bacterial species as they represent indigenous *Paenibacillus* sp. strains.

Interestingly, the phylogeny and comparative genomics analyses of this study suggested possible future taxonomic subdivision of the species *P. polymyxa*. The dendrograms based on ANI of *P. polymyxa* genomes and the maximum-likelihood tree based on the sequence homology of conserved genes of *P. polymyxa* genomes shared the same topology consisting of three major clades. Moreover, the comparative genomics analysis showed that the ANI values between strains from different clades were lower than 95%, which is the proposed prokaryotic species boundary^41,42^. While both Clade 1 and Clade 2 contained 18 *P. polymyxa* strains, the type strain of *P. polymyxa,* which is ATCC 842, was in Clade 2. There was no apparent patterns of geographic locations or environment origins of strains associated with each clade (Supplementary Table S3). Such results demonstrated that current *P. polymyxa* strains might need to be reclassified into three species based on ANI, including the “original” *P. polymyxa* species (Clade 2). The two novel *Paenibacillus* sp. strains (S02 and S25) are representatives of Clade 1 and represent a new *Paenibacillus* species. Similarly, the nine strains identified in Clade 3 also represent a new *Paenibacillus* species. Strains from the three clades were genetically closely related, and shared some PGP genes (e.g. IAA production)^16^ and secondary metabolite gene clusters (e.g. fusaricidin and tridecaptin, Table 1). However, the differences between strains from the three clades were demonstrated by the absence of some PGP genes from strains of a clade (e.g. the *nif* operon, Clade 3)^16^ and the presence of unique secondary metabolite gene clusters in strains of a clade (e.g. betalactone, Clade 2). Such phylogenomic reclassifications of *P. polymyxa* have been proposed in a previous study^43^. One of the reasons that caused this taxonomic ambiguity was the molecular markers used for taxonomic assignment. Prokaryotic taxonomy identification has been relying on the 16S ribosomal RNA over the last four decades, however the current standard is now shifting to genome-scale data^44^. For example, *P. polymyxa* E681 (Clade 1), which was isolated in 1990s and was the most studied *P. polymyxa* strain, was identified as *P. polymyxa* solely based on the 16S ribosomal RNA gene sequences^43^. However, the ANI analysis using its genome published in Kim, et al.^8^ only showed a similarity of 89.98% compared to the type strain *P. polymyxa* ATCC 842, whose genome was published in 2011^45^. Molecular phylogeny based on the 16S ribosomal RNA gene sequences led to the discovery of the novel genus *Paenibacillus*^46^, which now comprised more than 200 species and this number is still growing^4^. With more and more *Paenibacillus* spp. genomes becoming available, molecular taxonomy based on genome-scale data including ANI will clarify the complexity of *Paenibacillus* species.

### PGP genes and secondary metabolite gene clusters in the two *Paenibacillus* sp. strains

*P. polymyxa* has long been described as a PGP bacterium. The mode of action of plant growth promotion utilised by *P. polymyxa* has been proposed by Jeong, et al.^43^, including (1) direct promotion by producing phytohormones and enhancing nutrient uptake by plants, and (2) indirect promotion by providing bioprotection against phytopathogens. Method 1 is implemented via the expression of PGP genes. In this study, *Paenibacillus* sp. strains S02 and S25 were found to possess multiple PGP genes associated with biological nitrogen fixation, inorganic and organic phosphate solubilisation, phosphate assimilation and IAA production. Comparative genomics studies conducted by Eastman, et al.^16^ and Xie, et al.^5^ showed that these genes are highly conserved in *P. polymyxa* strains. For example, the *nif* operon carried by *P. polymyxa* strains contained nine genes and had a high sequence homology (> 80%)^4^. Interestingly, it has been reported that the ancestral *Paenibacillus* species could not fix atmospheric nitrogen, and the *nif* operon was acquired by N-fixing strains from possibly *Frankia* spp. via horizontal gene transfer^26^. Hence. these PGP genes were highly likely acquired by *Paenibacillus* spp. during the evolution to adapt to their plant-associated lifestyle^17^.

In this study, the two *Paenibacillus* sp. strains (S02 and S25) exhibited strong inhibitory activities against several fungal phytopathogens in in vitro assays. Such bioprotection (method 2) activities of *P. polymyxa* have been extensively studied. It has been discovered that *P. polymyxa* strains colonise plant roots and form biofilms, hence preventing the colonisation of phytopathogens^22^. Moreover, *P. polymyxa* strains also produce a wide variety of secondary metabolites associated with bioprotection. The two strains used in this study were found to carry 13 and 16 secondary metabolite gene clusters synthesising both antimicrobial ribosomal peptides, e.g. paenilan and paenicidin (lanthipeptide), and nonribosomal peptides including fusaricidin B, polymyxin and tridecaptin. Comparative analyses conducted in this study revealed that these clusters are conserved in *P. polymyxa* strains regardless of the phylogenetic clades. Furthermore, the compounds synthesised by more than half of the secondary metabolite gene clusters were novel, and they were commonly detected in many *P. polymyxa* strains^5,16,17^. These novel clusters represented a repository where more potential bioactive antimicrobial compounds could be discovered. Future research is required to identify and characterise these novel compounds.

Furthermore, some *P. polymyxa* strains have been reported to produce volatile compounds that promote plant growth by utilising both methods described above. For example, the volatile compounds emitted by *P. polymyxa* E681 increased the total leaf surface area of *Arabidopsis* seedlings and induced plant resistance to *Pseudomonas syringae*^47^. Thirteen volatile compounds produced by *P. polymyxa* WR-2 not only inhibited the growth of *F. oxysporum*, but also prevented the germination of *F. oxysporum* spores^48^. Hence, further studies are needed to identify and characterise bacterial volatile compounds produced by the novel *Paenibacillus* sp. strains S02 and S25.

### Transcriptome sequencing confirmed the activities of the *nif* operon and provided insights into the early stage bacteria-pathogen interactions

Transcriptome sequencing revealed that the presence/absence of nitrogen greatly affected the transcriptome profiles of the two *Paenibacillus* sp. strains (S02 and S25). The expression levels of all nine genes of the *nif* operon were regulated, and mostly increased, for both strains when nitrogen was removed from the medium. However, the level of changes of those genes caused by removing nitrogen varied between the two strains. There were much higher increases in expression levels of the *nif* genes in strain S02 when compared with strain S25. Given that the two strains shared similar expression levels of the *nif* operon before the removal of nitrogen, it could be concluded that strain S02 is more active in biological nitrogen fixation. Such increased bioactivity could be explained by the difference in growth kinetics. It has been found that strain S02 grows faster than strain S25 in both Burk’s medium and Nutrient Broth (based on OD_600_ readings after 24 h). It could be postulated that *Paenibacillus* sp. S02 requires more nitrogen to meet the demand of cell multiplication, which leads to increased bioactivities of the *nif* operon when nitrogen is removed from the growth medium. To validate this increased activity, further experiments using the acetylene reduction assay or similar methods^49^ are required to quantify the N-fixation rate of both strains.

The two *Paenibacillus* sp. strains (S02 and S25) were bioactive against *F. verticillioides* (growth reduction) in the in vitro assays, with strain S02 being significantly more bioactive than S25. Genomic analyses demonstrated that the two strains were capable of producing at least two antifungal secondary metabolite compounds, i.e. fusaricidin^50^ and bacterial siderophore^51^, as well as a wide range of novel secondary metabolite compounds. We proposed a hypothesis that these compounds were associated with the bioprotection activities against *F. verticillioides* exhibited by both strains. To support results from the bioassay and genomic analysis, a transcriptome sequencing experiment was designed to explore the early stage interactions between the two strains and the pathogen. The stronger bioprotection activities of strain S02 were reflected by the percentages of mapped *F. verticillioides* transcripts in the treated samples. One of the biological replicates of strain S02 even produced a mapping rate that was comparable to the untreated samples. Moreover, the treated samples of strain S02 were plated on Potato Dextrose Agar plates, and no visual evidence of fungal growth was observed for that biological replicate (Supplementary Figure S4). Such results suggested that, for this specific biological replicate, the growth of *F. verticillioides* was highly likely completely inhibited. Furthermore, the results of DGE analysis showed that the core biosynthetic genes of secondary metabolite gene clusters were actively expressed by strain S02 comparing to strain S25 before the introduction of *F. verticillioides*, including clusters producing two known antifungal compounds (C1: fusaricidin B; C2: bacterial siderophore) as well as clusters producing novel compounds (e.g. C6 and C14), which may have also contributed to the bioactivity. Hence, we proposed a hypothesis that the stronger bioprotection activities provided by strain S02 was related to the higher concentrations of antifungal bioactive compounds that were produced even before the introduction of *F. verticillioides.* In addition, changes in transcriptome profiles suggested that strain S02 was more resilient to the introduction of *F. verticillioides* when compared with strain S25. Such highly active and stable transcriptome profiles would make strain S02 a promising candidate to be developed as a bioprotection agent. Future experiments, including the in vitro and *in planta* assays and field assays, are needed to further validate the potential of this strain. The bioactive compounds produced by the two strains should also be identified, purified and characterised.

It is notable that 32 of 51 core biosynthetic genes of secondary metabolite clusters of strain S25 were differentially expressed when *F. verticillioides* was present, with 17 being downregulated including those from clusters producing known antifungal compounds. Given this strain was bioactive against *F. verticillioides* in the in vitro assays, a possible explanation was the incubation time. Whilst the two strains and *F. verticillioides* were co-incubated for five days in the in vitro bioassays, they were only co-incubated for six hours before RNA was extracted in the transcriptome sequencing experiment. The short incubation time may not be enough for strain S25 to produce significant amounts of antifungal compounds. It has been reported that the expressions of secondary metabolites of *Bacillus* spp. were enhanced when fungal pathogens (including some *Fusarium* spp.) were present^52^, however such information is still missing for *P. polymyxa* and *F. verticillioides*. It is possible that the decreased expressions of those genes observed in this study was related to the bacteria-pathogen interactions. Future studies should incorporate a longer incubation time or a series of time points, to further elucidate the changes in transcriptome profiles of the two strains after the introduction of *F. verticillioides.*

The two *Paenibacillus* sp. strains reported by this study were genetically highly similar (ANI = 97.78%) despite the apparent differences in bioactivities and transcriptome profiles. Interestingly, our laboratory has previously isolated and characterised three novel *Xanthomonas* sp. strains from the perennial ryegrass microbiome which were also genetically similar but phenotypically different^53^. Hence, we proposed a hypothesis that plant hosts recruit and take advantages of multiple genetically similar strains of the same species with a diverse range of bioactivities. Future studies, especially *in planta* assays, are required to further characterise those strains to gain a deeper understanding of the perennial ryegrass microbiome.

## Materials and methods

### Seed-associated N-fixing bacterial strain detection and isolation

A PCR assay was designed to detect the presence of seed-associated N-fixing bacteria by amplifying the *nifH* gene of nitrogenase. Perennial ryegrass seeds (*L. perenne*, cv. Alto, with standard endophytes) were sourced from Barenbrug Agriseeds, New Zealand with licences that comply with local and national regulations. Approximately 1,000 seeds were washed using sterile water and then ground and soaked in 30 mL of Burk’s N-free medium (MgSO_4_, 0.2 g/L; K_2_HPO_4_, 0.8 g/L; KH_2_PO_4_, 0.2 g/L; CaSO_4_, 0.13 g/L; FeCl_3_, 0.00145 g/L; Na_2_MoO_4_, 0.000253 g/L; sucrose, 20 g/L). The suspension was incubated for two days at 26 ℃ and 200 rpm, and then serial diluted using sterile Burk’s N-free medium (1:10, 100 µL in 900 µL, eight replicates per dilution). Genomic DNA was extracted from the 10^–2^ and 10^–3^ dilutions using a Wizard Genomic DNA Purification Kit (A1120, Promega, Madison, WI, USA), and assessed for quality on a NanoDrop 2000 (Thermo Scientific, Waltham, MA, USA). PCR conditions were as per Gaby and Buckley^54^. In brief, OneTaq^@^ Hot Start 2 × Master Mix (M0484, Promega, Madison, WI, USA) was used with a universal *nifH* gene PCR primer pair (IGK3: 5’- GCIWTHTAYGGIAARGGIGGIATHGGIAA-3’; DVV: 5’-ATIGCRAAICCICCRCAIACIACRTC-3’; final concentration = 0.4 µM)^54^ and 50 ng of extracted DNA. For the no template control, nuclease-free water was used. For the positive control, the genomic DNA of *Rhizobium leguminosarum* bv. *trifolii* WSM1325^55^ was used. PCR products (~ 400 bp) were visualised on an Agilent 2200 TapeStation (Agilent Technologies, Santa Clara, CA, USA), sequenced by Macrogen and then analysed using BLAST^56^.

Genomic DNA of dilutions that produced PCR amplicons were sequenced using long read sequencing technology. A library was prepared using the Oxford Nanopore Technologies (ONT) ligase-based library preparation kit (SQK-LSK109, ONT, Oxford, UK) and sequenced on a MinION Mk1B platform (MIN-101B) with R10 flowcells (FLO-MIN110). Genomic sequence data (raw read signals) were basecalled using ONT’s Guppy software (Version 3.4.3, HAC basecalling model), and assessed for quality using NanoPlot^57^. Basecalled data was filtered to remove adapter sequences using Porechop (Version 0.2.3, https://github.com/rrwick/Porechop), while reads shorter than 300 bp and the worst 5% of reads (based on quality) were discarded using Filtlong (Version 0.2.0, https://github.com/rrwick/Filtlong). Sequencing reads were taxonomically classified by Kraken2^24^ using a custom database containing all completed bacterial reference genomes in NCBI (20/03/2020) and were analysed using BLAST^56^. In addition, 50 µL of those dilutions were inoculated into vials containing 5 mL of Burk’s N-free medium supplemented with 1.6 g/L agar and incubated for up to five days at 26 ℃. Cultures were checked daily for a band of microbial growth below the surface of medium, which indicated the presence of N-fixing bacteria^38^. Microbes were streaked onto Burk’s N-free medium supplemented with 15 g/L agar and Burk’s N-free medium supplemented with 15 g/L agar and 100 IU/mL polymyxin B (P4932-1MU, Sigma-Aldrich, St. Louis, MO, USA) and incubated for up to five days at 26 ℃ to isolate pure colonies.

### Genome sequencing

DNA was extracted from bacterial pellets (overnight cultures) using a Wizard Genomic DNA Purification Kit (A1120, Promega, Madison, WI, USA), and assessed for quality (average molecular weight ≥ 30 Kb) on an Agilent 2200 TapeStation (Agilent Technologies, Santa Clara, CA, USA). Genomic sequencing libraries (short reads) were prepared from the DNA using a PerkinElmer NEXTFLEX Rapid XP DNA-Seq Kit (Cat# NOVA-5149-03) and sequenced on an Illumina NovaSeq 6000 platform. Genomic sequence data (raw reads) were assessed for quality and filtered to remove any adapter and index sequence, and low-quality bases using fastp^58^ with the following parameters: *-w 8 -3 -5 -detect_adapter_for_pe*. In addition, genomic sequencing libraries (long reads) were prepared from the DNA as per “Seed-associated N-fixing bacterial strain detection and isolation” section.

### Genome assembly and classification

The whole genomes of bacterial strains were assembled with filtered long and short reads using Unicycler^59^. Long reads were used for primary assembly and to resolve repeat regions in the genome, whereas short reads were used to correct small base-level errors. Assembly graphs were visualised using Bandage^60^. Assembled genomes were taxonomically classified by Kraken2^24^ as per “Seed-associated N-fixing bacterial strain detection and isolation” section.

### Genome annotation and characterisation

The assembled genomes of bacterial strains were annotated using Prokka^61^ with a custom *Paenibacillus* protein database (based on Kraken2 classification) to predict genes and corresponding functions. Identification of secondary metabolite gene clusters from annotated genomes was conducted using antiSMASH^32^ with the following options: *--clusterblast --asf --knownclusterblast --subclusterblast --smcogs --full-hmmer*. The presence of PGP genes in the annotated genomes was conducted using BLAST^56^ (blastn and tblastn, e value > 1e^−10^). PGP genes previously reported in *P. polymyxa* strains^5,16^ were targeted (30 genes), including biological nitrogen fixation (nine genes), phosphate solubilisation and assimilation (17 genes) and indole-3-acetic acid production and auxin transportation (four genes). The PGP gene identification compared the sequence homology of genes from strains S02 and S25 with a closely related *P. polymyxa* strain CR1.

### Phylogeny and comparative genomics

A comparative genomic analysis was performed by calculating the average nucleotide identity (ANI) of the genomes of isolated strains to 44 *P. polymyxa* genomes that were publicly available on NCBI (Supplementary Table S3) using a python package pyani (Version 0.2.8, https://widdowquinn.github.io/pyani/). Moreover, a pan-genome analysis was conducted using Roary^27^ to compare the isolated strains to 13 *P. polymyxa* strains with complete circular genome sequences (Fig. 1, yellow labels) and to identify shared genes. A maximum-likelihood phylogenetic tree was inferred using FastTree^62^ with Jukes-Cantor Joins distances, the Generalized Time-Reversible substitution model and the CAT approximation model. Local branch support values were calculated using 1000 resamples with the Shimodaira-Hasegawa test.

### Bioprotection assay (in vitro)

An assay was conducted to assess the in vitro bioprotection activity of isolated strains against fungal pathogens of *Poaceae* species. Three fungal pathogens of *Poaceae* species (Supplementary Table S9) were obtained from the National Collection of Fungi (VPRI, Bundoora, Victoria, Australia). The setup of the in vitro assay was described in detail in Li, et al.^53^. Briefly, bacterial strains, which were drop-inoculated onto four equidistant points on a Nutrient Agar plate, and pathogens, which were placed at the centre of the plate as a plug containing actively growing hyphae, were co-incubated at 28 ℃ in the dark for five days. The diameter of the fungal colony was measured twice, and the average of the two readings was used for statistical analysis. Three plates were prepared for each treatment as biological replicates. Sterile medium was used as the blank control. Statistical analysis (One-way ANOVA and Tukey Test) was conducted using OriginPro 2020 (Version SR1 9.7.0.188) to detect the presence of any significant difference (*P* < 0.05) between treatments.

### Transcriptome sequencing

Transcriptome sequencing experiments were designed to confirm the expression of the *nif* operon and to explore the early stage of bacteria-pathogen interactions. For the N-fixation activity assay, bacterial strains were cultured in Burk’s N-free medium overnight (OD_600_ = 1.0). Cultures were diluted using Burk’s N-free medium to OD_600_ = 0.7 and further cultured for six hours to produce actively growing cells for extracting high quality RNA. Burk’s N-free medium supplemented with 10 g/L NH_4_Cl was used for culturing the bacterial strains as the control. For the bacteria-pathogen interactions assay, bacterial strains and the phytopathogen VPRI42586a *Fusarium verticillioides* were cultured in Nutrient Broth overnight (OD_600_ = 1.0). Bacterial cultures were diluted using Nutrient Broth to OD_600_ = 0.7. 20 mL of such culture was mixed with 200 µL of the pathogen culture and was further incubated for six hours. For the control, the pathogen culture was replaced by sterile Nutrient Broth. Three biological replicates were prepared for each treatment.

Total RNA was extracted form cell pellets using a TRIzol Plus RNA Purification Kit (12183555, Thermo Fisher Scientific). On-column treatments were conducted using a PureLink DNase Kit (12185010, Thermo Fisher Scientific) to ensure the complete removal of genomic DNA that would affect the downstream analyses, and ribosomal RNA was depleted using a NEBNext rRNA Depletion Kit (E7860L, NEB, Ipswich, MA, USA). Directional RNA-seq libraries were prepared using a NEBNext Ultra II Directional RNA Library Prep Kit (E7765) and sequenced on an Illumina NovaSeq 6000 platform. RNA-seq data (raw reads) were assessed for quality and filtered as per described in “Genome sequencing” section.

### Transcriptome analysis

Salmon^63^ was used to quantify transcripts using the clean RNA-seq reads with the following parameters: *-l A --validateMappings --numBootstraps 1000 --seqBias*. The references used for transcript quantification were the gene sequences generated by Prokka (“Genome annotation and characterisation” section) or the gene sequences of *F. verticillioides* 7600 downloaded from NCBI GenBank (Accession ID: SAMN02953630). A total of 1,000 rounds of bootstraps were performed during transcript quantification to minimise the impact of technical variations. Differential gene expression (DGE) analysis was conducted using a R package sleuth^64^. Likelihood ratio tests were conducted to detect the presence of any significant difference (*q-value* < 0.05) in transcript abundances between treatments, and Wald tests were conducted to determine an approximation of the fold-change in transcript abundances between treatments. Transcripts that were of ultra-low abundance (defined by having less than 20 mapped reads or were only present in less than three samples) were removed prior DGE analysis. The differentially expressed genes were defined to be significant at *q-value* < 0.05 and absolute fold-change ≥ 1.5.

## Supplementary Information

Supplementary Information 1.

Supplementary Information 2.

Supplementary Information 3.

Supplementary Information 4.

## Data Availability

Annotated genome sequences of strains S02 and S25 were deposited in the NCBI GenBank with the accession number PRJNA720481. The results of transcript quantification (raw reads count) were provided as supplementary datasets (Supplementary Table S10 and S11).
