# Supplementary material for: Transcriptomics differentiate two novel bioactive strains of Paenibacillus sp. isolated from the perennial ryegrass seed microbiome
Source: Sci Rep. 2021 Jul 30;11:15545. doi: 10.1038/s41598-021-94820-2 (PMC8324883; doi:10.1038/s41598-021-94820-2)
Supplement: Supplementary file 1 — Supplementary Information 1. [file 41598_2021_94820_MOESM1_ESM.pdf]

Supplementary Table S1. Summary of reads available for genome assembly

Strain ID	Yield (bp) Illumina	Yield (bp) ONT	Mean length (bp) ONT	Coverage Illumina	Coverage ONT
S02	1,555,571,650	8,073,984,100	1,144	256.7×	1332.2×
S25	981,251,546	5,129,702,300	5,088	164.7×	860.8×

Supplementary Table S2. General genomic characteristics of *Paenibacillus* sp. strains S02 and S25

Strain ID	Genome size (bp)	GC content (%)	No. of tRNA	No. of tmRNA	No. of rRNA	No. of gene	No. of CDS
S02	6,060,529	45.60	92	1	33	5,436	5,310
S25	5,958,851	45.72	92	1	36	5,306	5,177

Supplementary Table S4. Plant growth-promoting genes in the genomes of *Paenibacillus* sp. strains S02 and S25, and *P. polymyxa* strain CR1

Trait	Gene Name	*Paenibacillus* sp. S02	*Paenibacillus* sp. S25	*P. polymyxa* CR1
Nitrogen fixation				
	nifB	KAI36_01039	KAI37_00991	YP_008910495
	nifH	KAI36_01040	KAI37_00992	YP_008910496
	nifD	KAI36_01041	KAI37_00993	YP_008910497
	nifK	KAI36_01042	KAI37_00994	YP_008910498
	nifE	KAI36_01043	KAI37_00995	YP_008910499
	nifN	KAI36_01044	KAI37_00996	YP_008910500
	nifX	KAI36_01045	KAI37_00997	YP_008910501
	hesA/moeB	KAI36_01046	KAI37_00998	YP_008910502
	nifV	KAI36_01047	KAI37_00999	YP_008910503
Phosphate solubilization				
	gcd	KAI36_02793	KAI37_02644	YP_008912273
	gad	–	–	–
Phosphonate cluster (*phn*)				
	phnA	KAI36_05309	KAI37_05177	YP_008914717
	phnB	KAI36_00880	KAI37_00854	YP_008910326
	phnC	KAI36_04607	KAI37_04494	YP_008913947
	phnD	KAI36_04606	KAI37_04493	YP_008913946
	phnE	KAI36_04608	KAI37_04495	YP_008913948
	phnW	KAI36_05275	KAI37_05152	YP_008914692
	phnX	KAI36_00485	KAI37_00496	YP_008909947
	ppd	KAI36_05276	KAI37_05153	YP_008914693
	pepM	KAI36_05277	KAI37_05154	YP_008914694
Phosphate transporter (*pst*)				
	pstS	KAI36_01689	KAI37_01595	YP_008911198
	pstA	KAI36_01691	KAI37_01597	YP_008911200
	pstB	KAI36_01692	KAI37_01598	YP_008911201
	pstC	KAI36_01690	KAI37_01596	YP_008911199
	phoP	KAI36_01703	KAI37_01609	YP_008911212
	phoR	KAI36_01702	KAI37_01608	YP_008911211
Indole-3-acetic acid production				
	ipdC	KAI36_01475	KAI37_01435	YP_008911027
	auxin efflux carriers	KAI36_02845<br>KAI36_03330<br>KAI36_05253	KAI37_02683<br>KAI37_03351<br>KAI37_05129	YP_008912813<br>YP_008912323<br>YP_008911849

Gene identifiers for *Paenibacillus* sp. strains S02 and S25 were from the annotated genomes described in section 2.2. Gene identifiers for *P. polymyxa* CR1 were from Eastman, et al.[16]

Supplementary Table S5. Percentage identity of plant growth-promoting genes in the genome of strains *Paenibacillus* sp. S02/S25 and *P. polymyxa* CR1

**Trait**	**Gene Name**	**S02 VS S25**	**S02 VS CR1**	**S25 VS CR1**
Nitrogen fixation				
	nifB	97.13	98.00	96.80
	nifH	95.39	98.15	94.69
	nifD	98.96	98.62	98.27
	nifK	98.24	97.45	97.39
	nifE	98.24	98.60	98.16
	nifN	97.25	97.55	97.63
	nifX	98.46	97.69	97.69
	hesA/moeB	96.47	98.04	96.34
	nifV	98.50	98.59	97.80
Phosphate solubilization				
	gcd	98.11	97.60	98.39
	gad	-	-	-
Phosphonate cluster (*phn*)				
	phnA	98.53	98.53	98.82
	phnB	98.21	98.43	99.78
	phnC	97.66	97.81	98.39
	phnD	98.14	99.07	97.63
	phnE	98.25	97.78	97.43
	phnW	98.74	98.47	98.20
	phnX	98.04	97.92	98.81
	ppd	98.97	98.62	98.79
	pepM	99.33	99.44	99.00
Phosphate transporter (*pst*)				
	pstS	98.92	98.48	99.13
	pstA	96.99	97.55	98.22
	pstB	98.81	98.70	98.22
	pstC	99.14	98.71	98.82
	phoP	98.09	97.81	97.94
	phoR	98.28	97.45	97.89
Indole-3-acetic acid production				
	ipdC	99.54	99.48	99.14
	auxin efflux carriers 1	99.13	98.15	98.37
	auxin efflux carriers 2	97.92	97.60	98.85
	auxin efflux carriers 3	98.58	98.96	99.05

Supplementary Table S6. Secondary metabolite gene clusters identified in *Paenibacillus* sp. strains S02 and S25

**ID**	**Type**	**Location**		**Most similar known cluster (similarity)**
		**S02**	**S25**	
C1	Nrps	62,712-130,949	62,863-131,149	**fusaricidin B (100%)**
C2	siderophore	1,060,830-1,078,231	1,021,525-1,038,926	–
C3	bacteriocin	1,226,685-1,236,921	1,163,825-1,174,061	–
C4	Nrps<br>transAT-PKS	1,276,170-1,374,849	1,234,262-1,333,113	–
C5	lassopeptide	1,410,732-1,434,848	1,369,083-1,393,199	paeninodin (40%)
C6	Nrps	1,496,857-1,557,694	1,452,621-1,513,241	marthiapeptide A (33%)
C7	lanthipeptide	1,752,471-1,779,477	1,717,117-1,742,305	**paenilan (100%)**
C8	lanthipeptide	–	1,865,337-1,891,786	**paenicidin B (71%)**
C9	Nrps-like	2,147,919-2,191,265	2,068,326-2,110,857	–
C10	Nrps	2,564,994-2,657,512	2,538,234-2,631,151	**tridecaptin (100%)**
C11	Nrps<br>transAT-PKS	2,800,573-2,881,430	2,762,852-2,843,624	paenilipoheptin<br>(S02, 73%; S25, 76%)
C12	Nrps	–	2,847,977-2,929,143	–
C13	Nrps<br>betalactone	–	3,004,139-3,056,727	–
C14	Nrps<br>T3PKS<br>transAT-PKS	3,755,116-3,856,856	3,756,437-3,858,120	aurantinin B/C/D (35%)
C15	Nrps	5,189,939-5,270,981	5,092,876-5,173,931	**polymyxin (100%)**
C16	phosphonate	5,879,383-5,920,282	5,775,191-5,816,090	–

Nrps: Nonribosomal peptide synthetase
transAT-PKS: transAT-polyketide synthase
T3PKS: Type III polyketide synthase
Clusters in bold: Known antimicrobial compounds

Supplementary Table S7. The average colony diameter (± standard error) of fungal pathogens when exposed to the two *Paenibacillus* sp. strains (S02 and S25) in a bioprotection assay (*in vitro*)

**Pathogen ID**	**S02/cm**	**S25/cm**	**Blank/cm**
Colletotrichum graminicola	1.03 ± 0.02^{b}	3.77 ± 0.04^{a}	4.10 ± 0.20^{a}
Fusarium verticillioides	2.93 ± 0.04^{c}	6.15 ± 0.14^{b}	6.80 ± 0.08^{a}
Microdochium nivale	5.12 ± 0.11^{a}	5.18 ± 0.12^{a}	5.27 ± 0.04^{a}

$^{a, b, c}$: Different letters are statistically significantly ($P < 0.05$) different.
S02/S25: *Paenibacillus* sp. strains

Supplementary Table S8. The percentage of mapped reads when quantifying transcript of each sample using the transcriptome sequences of *F. verticillioides* 7600 as the reference

**Sample**	**No. of mapped reads**	**Mapping rate**
S02_ctrl_1	4	too low
S02_ctrl_2	1	too low
S02_ctrl_3	2	too low
S02_treated_1	3	too low
S02_treated_2	1,492	0.0082%
S02_treated_3	1,366	0.0089%
S25_ctrl_1	1	too low
S02_ctrl_2	0	0%
S02_ctrl_3	0	0%
S25_treated_1	33,944	0.2817%
S25_treated_2	17,987	0.1407%
S25_treated_3	22,684	0.1395%

too low: 0–0.0001%; ctrl: Without *F. verticillioides*; treated: With *F. verticillioides*
S02/S25: *Paenibacillus* sp. strains isolated in this study

Supplementary Table S9. Pathogens used in the *in vitro* bioprotection assay.

**VPRI Acc. No.**	**Taxonomic Details**	**Host Taxonomic Details**	**State**	**Collection Date**
32315	*Colletotrichum graminicola*	*Cynosurus echinatus.*	Vic.	27-Apr-05
42586a	*Fusarium verticillioides*	*Zea mays* L.	Vic	27-Feb-15
43403	*Microdochium nivale*	*Lolium perenne*	Vic.	11-Nov-17

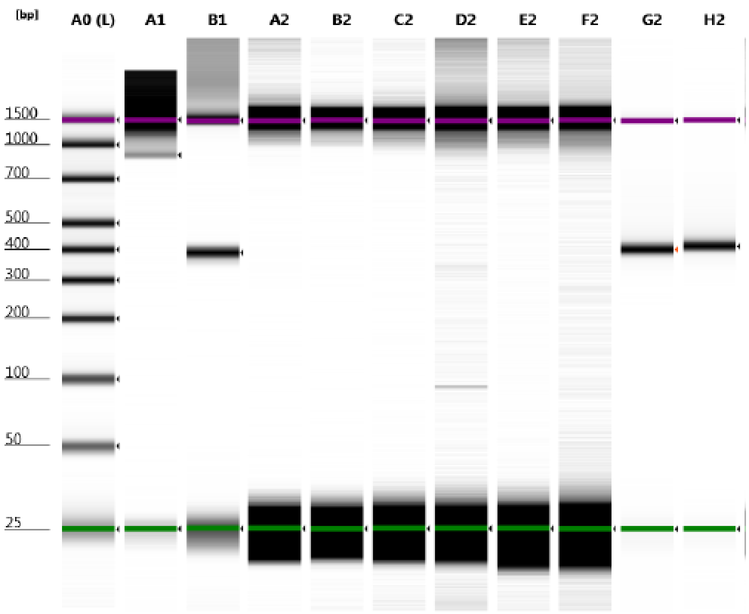

Supplementary Figure S1. Amplicons of the expected size (~ 400 bp) were produced using the DNA extracted from two (G2 and H2) of eight replicates of the 10^{-2} dilution (A2–H2). Results of the 10^{-3} dilutions were not shown since no amplicon was produced.
A0: DNA ladder; A1: No template control; B1: Positive control, *Rhizobium leguminosarum* bv. *trifolii* WSM1325 DNA

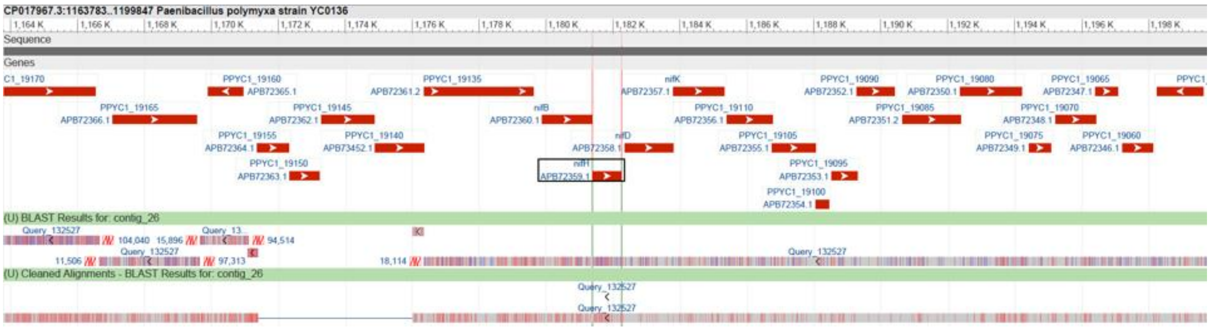

Supplementary Figure S2. A single 110 Kb read containing the entire *P. polymyxa nif* operon (nine genes) of *P. polymyxa* YC0136 identified via BLAST analyses. The *nifH* gene was highlighted.

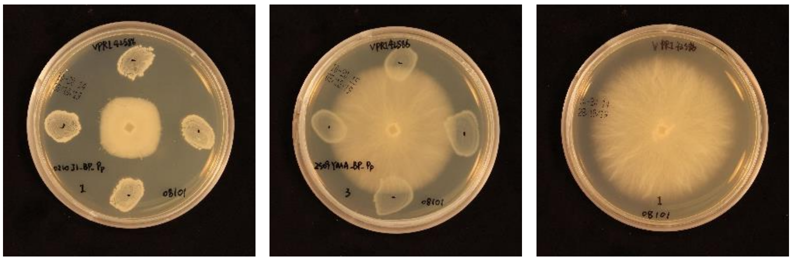

Supplementary Figure S3. Representative images of the in vitro bioprotection assay when challenging strain S02 (left) and S25 (middle) with *Fusarium verticillioides*. The control plate (pathogen-only) was shown on the right.

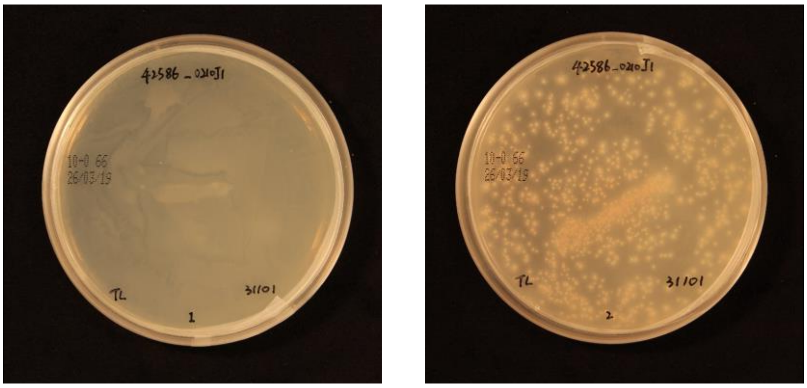

Supplementary Figure S4. Images of Potato Dextrose Agar plates containing samples of *Paenibacillus* sp. S02 co-incubated *F. verticillioides* (six hours). The first biological replicate (S02_treated_1, left) showed no visual growth of the pathogen, and only had three mapped fungal reads. The second biological replicate (S02_treated_2, right) had 1,492 mapped fungal reads.
